# Beyond the Extra Respiration of Phagocytosis: NADPH Oxidase 2 in Adaptive Immunity and Inflammation

**DOI:** 10.3389/fimmu.2021.733918

**Published:** 2021-09-01

**Authors:** Paige M. Mortimer, Stacey A. Mc Intyre, David C. Thomas

**Affiliations:** Centre for Inflammatory Disease, Department of Immunology & Inflammation, Imperial College, London, United Kingdom

**Keywords:** NOX2, ROS, CGD, oxidative stress, systemic inflammation

## Abstract

Reactive oxygen species (ROS) derived from the phagocyte NADPH oxidase (NOX2) are essential for host defence and immunoregulation. Their levels must be tightly controlled. ROS are required to prevent infection and are used in signalling to regulate several processes that are essential for normal immunity. A lack of ROS then leads to immunodeficiency and autoinflammation. However, excess ROS are also deleterious, damaging tissues by causing oxidative stress. In this review, we focus on two particular aspects of ROS biology: (i) the emerging understanding that NOX2-derived ROS play a pivotal role in the development and maintenance of adaptive immunity and (ii) the effects of excess ROS in systemic disease and how limiting ROS might represent a therapeutic avenue in limiting excess inflammation.

## 1 Introduction

### 1.1 Reactive Oxygen Species

Reactive Oxygen Species (ROS) are small molecules that are derived from molecular oxygen. They can either be classed as radicals or non-radicals, depending on whether they have an unpaired electron (1). Superoxide (a radical) is typically restricted to the endosomal compartment, and can be converted into hydrogen peroxide (H_2_O_2_; a non-radical) at low pH. H_2_O_2_ can diffuse across membranes to oxidise specific targets, or can be converted to O_2_ and H_2_O (2).

H_2_O_2_ is a very useful signalling molecule because it can be rapidly generated and rapidly removed *via* specific enzymes such as catalase, superoxide dismutase and peroxiredoxin enzymes. It can also be quenched by non-enzymatic means such as glutathione (GSH) (3). As such, by predominantly facilitating cysteine and methionine oxidation, H_2_O_2_ is integral to regulating several crucial facets of the immune response.

ROS are produced during metabolic reactions within many cellular compartments, including the mitochondria, peroxisome and endoplasmic reticulum (4, 5). This review, however, will focus on ROS specifically produced in the phagosomes and at the cell membrane, by the phagocyte NADPH oxidase NOX2. ROS generation can occur from many sources in cells. These include mitochondria, peroxisomes and the P450 enzyme system. The NADPH oxidase is the first example of an enzyme where generating ROS is the primary function of the system, not a by-product of another process, e.g. the generation of ATP in mitochondria [discussed in (6)].

### 1.2 A Brief History of ROS Discovery

The physiological production of ROS was first described in 1908, by the German biochemist Otto Warburg, who identified that following the fertilisation of sea urchin eggs, H_2_O_2_ production succeeded a large and rapid increase in oxygen consumption (7). He suggested the existence of a respiratory enzyme that utilised oxygen to generate ROS, for which he won the Nobel Prize in Physiology and Medicine (8).

The ability of phagocytes to produce ROS was first noted by Baldridge and Gerrard in 1933 who described a marked increase in oxygen uptake by canine neutrophils following phagocytosis (9). Sbarra and Karnovsky extended these findings to show that “this burst of extra respiration” was accompanied by glucose consumption *via* the hexose monophosphate shunt and lactate production (10). Crucially, inhibitors of mitochondrial respiration have no effect on the oxygen consumption that accompanies phagocytosis. This is because the purpose of the oxygen consumption is independent from aerobic glycolysis, and is instead required to generate ROS.

Further key milestones followed, including (i) the finding that NADPH is the dominant physiological electron donor (although both NADH and NADPH can act in this capacity) that allows the production of ROS (11–13) and (ii) the seminal observation that the process starts with the generation of superoxide (14).

These findings show that neutrophils possess an enzyme that facilitates the donation of electrons to molecular oxygen. The identification of cytochrome b558, which we refer to as NOX2, as the relevant enzyme resulted from insightful biochemistry and the study of the monogenic immunodeficiency X-linked chronic granulomatous disease (X-CGD). This “fatal granulomatous disease of childhood” was first described in the 1950s. It described boys whose neutrophils were unable to kill certain bacteria and did not increase oxygen consumption or produce ROS (15).

In a landmark study for the field, Segal and colleagues showed that neutrophils from patients with CGD lacked both NADPH oxidase activity and a particular unusual b type cytochrome that localised to the plasma membrane (16, 17). The suspected causative genetic region was localised to Xp21 and cloned (18). The cDNA identified from such studies was used to make a translated protein and an anti-serum was raised to it. Elegant studies showed that the anti-sera stained a 91kDa protein found in “purified cytochrome b558” preparations. Crucially, it could not stain neutrophils from patients with X-CGD (19). Thus, the unusual cytochrome identified by Segal was indeed the product of the gene that was disrupted in X-CGD. However, it was clear that the story was not quite that simple. For instance (i) the 91kDa membrane-bound protein transcribed and translated from the X chromosome co-purified with a 22kDa protein (20, 21) and (ii) it transpired that there were autosomal recessive forms of CGD (AR-CGD) associated with a deficiency of other specific proteins (22, 23).

### 1.3 Chronic Granulomatous Disease

**Figure 1:**
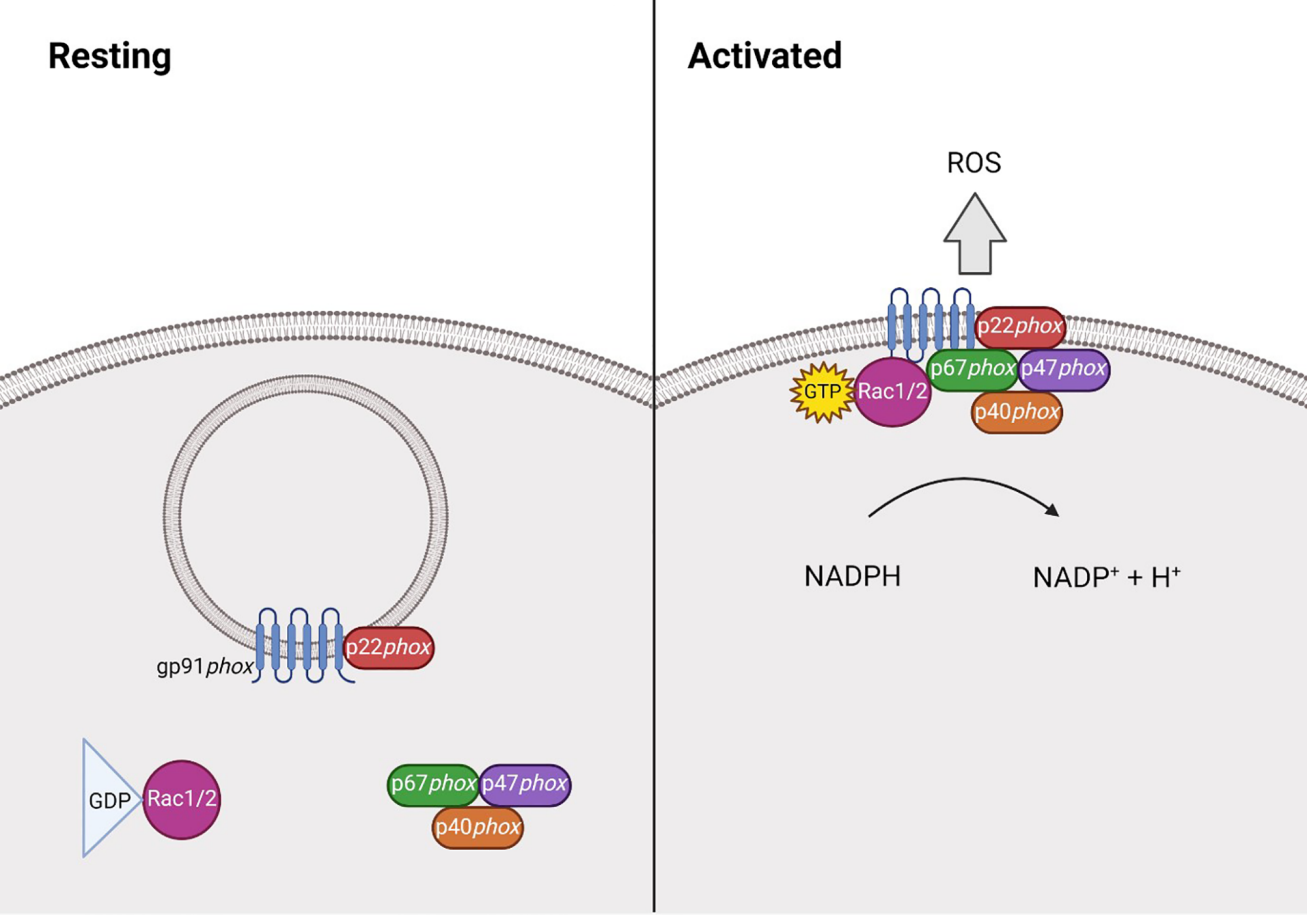
Structure of NADPH oxidase 2 during resting and activated states. At rest NADPH oxidase 2 (NOX2) is comprised of the membrane bound heterodimer gp91*phox-*p22*phox*, and the cytosolic component, comprised of p40*phox* (24), p47*phox* (22), p67*phox* (22) and the GTPase Rac1 (25) or Rac2 (26). Following stimulation, p47*phox* becomes phosphorylated and the Rac protein becomes GTP-bound, allowing the complex to translocate to the membrane where it can associate with the gp91*phox*-p22*phox* heterodimer. Together, this forms the activated complex that generates reactive oxygen species (ROS) by transferring electrons from NADPH.

The phagocyte NADPH oxidase (NOX2) is a multi-subunit protein complex that, upon interaction, can form an active enzyme complex capable of producing superoxide. It is comprised of two integral membrane bound components; the 91kDa gp91*phox* and 22kDa p22*phox*, which together form cytochrome b558 (16, 21). p22*phox* binds to and stabilises gp91*phox*, preventing its degradation and its own in return. The cytosolic components comprise of p40*phox* (24), p47*phox* (22), p67*phox* (22) and Rac1 (25) or Rac2 (26). Following stimulation, p47*phox* becomes phosphorylated, allowing the complex to translocate to the membrane where it can associate with the gp91*phox*-p22*phox* heterodimer, forming the activated complex that transfers electrons from NADPH to molecular oxygen (**Figure 1**). This process is known as the respiratory burst, which is essential during the innate immune response (27–30). ROS can also be generated by the other NOX family members, NOX1, NOX3, NOX4, NOX5, DUOX1 and DUOX2 (28), however this review will focus on NOX2 derived ROS.

Our understanding of CGD has improved as several large-scale cohort studies have been conducted. X-linked CGD is predominant in Europe (31–33), the United States (34) and Japan (35), accounting for approximately 60% of cases. p47*phox* deficiency accounts for around 30% of cases and p22*phox* and p67*phox* deficiency for the remaining 10%. AR-CGD is predominant in cohorts from countries such as Iran (36) or Turkey (37), where consanguineous marriage is more prevalent.

X-CGD patients have a more severe disease course than patients with the AR-CGD, presumably because they lack all oxidase activity (31). Residual oxidase activity in neutrophils is linked to reduced disease severity and modest production of ROS seems to confer a greater likelihood of long-term survival.

### 1.4 Anti-Microbial Action of NOX2-Derived ROS

The role of NOX2-derived ROS was first identified in killing microbes. This is well illustrated by the susceptibility of patients with CGD to an extensive, but nonetheless demarcated, range of pathogens. For example, neutrophils from patients with CGD can kill *E. coli* but not *S. Aureus*. The focus of this review is the extra-phagosomal role of ROS, but it is worthwhile describing briefly how neutrophil-mediated killing takes place. This can be both direct, where hydrogen peroxide is likely more microbicidal than superoxide, but also indirect through integration with other systems. Here reactive oxygen species collaborate with granule proteins such as myeloperoxidase (MPO). In the presence of H_2_O_2_, MPO catalyses the production of Hypochlorous acid (HOCL), a very potent anti-microbial agent. HOCL, in turn, can modify multiple proteins (both host and microbe) in the phagosome to generate chloramines and aldehydes.

While we do not cover these processes in detail, we would direct readers interested in this fascinating area to excellent reviews by Nauseef (38) and also, Winterbourn and Kettle (39). These expand more on a few key points:

(i) That there is synergy between ROS and other granule constituents, including HOCL and non-oxidative killing mechanisms such as cathelicidins, serine proteases and lactoferrin.
(ii) That microbes employ a variety of strategies to evade phagosomal killing.
(iii) That though the phagocyte NADPH oxidase is present in both neutrophils and macrophages, the intra-phagosomal environment in these cells differs greatly with respect to parameters such as pH and other anti-microbial components (such as MPO).

### 1.5 EROS Regulates the Expression of NOX2

An important recent addition to the biology of the phagocyte NADPH oxidase and CGD is the discovery of EROS (gene symbol CYBC1) (40). In 2017, we demonstrated that mice deficient in the previously uncharacterised open reading frame, bc017643, were exquisitely susceptible to infection with *Salmonella* Typhimurium and *Listeria monocytogenes*. It transpired that they failed to make ROS, owing to almost complete deficiency of gp91*phox* and p22*phox*. bc017643 encodes an ER-resident transmembrane protein that co-immunoprecipitates with gp91*phox*. We also observed that P2X7 receptor expression was downregulated in EROS deficient cells, which was later independently verified by another group (41), demonstrating that EROS mediates the expression of both gp91*phox* and P2X7. We characterised the protein encoded by bc017643 as EROS (Essential for Reactive Oxygen Species). Recent work in our laboratory suggests that EROS is a highly selective placeholder chaperone, binding to an “unsatisfied surface” on the gp91*phox* precursor and stabilising it until p22*phox* can bind (Randzavola, Mortimer et al., submitted). The lack of ROS and NOX2 expression in EROS-deficient mice, akin to that of gp91*phox*-/- mice, suggested that mutations in the human orthologue *C17ORF62* might lead to CGD. In 2018, we and another group reported separate homozygous mutations in *C17ORF62*, leading to EROS-deficiency, as a novel cause of chronic granulomatous disease (CGD5) (42, 43). *C17ORF62* has been re-named *CYBC1* (CYtochrome B Chaperone 1).

Mutations in EROS represent the first type of CGD to arise as a result of altered stability or folding of gp91*phox*. Although they have not yet been implicated in CGD, several other proteins are capable of regulating gp91*phox* abundance. Negative Regulator of Reactive Oxygen Species (NRROS) was described in 2014 as an ER-resident protein that binds gp91*phox* and facilitates its degradation (44), its role appearing opposite to that of EROS. Similarly, the heat shock proteins, hsp90 and hsp70, also have opposing effects on gp91*phox* abundance, stabilising and degrading it respectively (45). The lamin beta receptor (LBR) has an intriguing role in regulating gp91*phox* and neutrophils that are LBR deficient show lower expression of gp91*phox* and generation of zymosan-induced ROS (46, 47).

Regulating gp91*phox* is one method of regulating ROS production. It is essential to tightly control the levels of ROS and subsequent sections of this review examine the tissue damage that can be caused by excess ROS production. However, too little ROS can also lead to inflammation. A key observation from studying CGD is that patients do not only experience opportunistic infections, but also present with autoinflammatory and autoimmune manifestations. These manifestations are characterised by sterile granulomatous inflammation, a hallmark of CGD (48). CGD patients often develop autoimmune diseases in which the pathogenesis is driven by autoantibody production, such as systemic lupus erythematosus and juvenile rheumatoid arthritis (31, 34). By producing H_2_O_2_, the phagocyte NAPDH oxidase regulates multiple pathways involved in innate anti-microbial defence, often serving to restrain inflammation in the process.

### 1.6 NOX2 Regulates Inflammation and Immune Signalling

The inflammatory manifestations that affect CGD patients arise as loss of ROS signalling impairs type 1 interferon signalling and autophagy (29). Patients with X-CGD are between 50-90% more likely to experience inflammatory episodes compared to patients with AR-CGD (49, 50), suggesting NOX2, particularly gp91*phox*, is essential for controlling the balance between a successful immune response and tissue damage.

## 2 NOX2 in Regulating Processes in Antigen Presentation

The regulation of type 1 interferon signalling, inflammasome activation and autophagy is well documented in the innate immune system and we have covered this in previous reviews (29, 30). The role of ROS in adaptive immunity starts with its pivotal role in antigen presentation.

### 2.1 MHC Class I Processing and Presentation

In normal circumstances, ROS generation occurs concomitantly with phagocytosis, regardless of cell type. Superoxide is a weak base and tends to alkalinise the phagosome, which influences proteolysis. A key study showed that the normal pH of dendritic cells (DCs) is neutral, tending towards slightly alkaline, but that NOX2-deficient DCs have acidic phagosomes which leads to enhanced antigen degradation and impaired cross presentation to CD8+ T cells *via* MHC Class I (51). The same group reported similar results in human DCs (52) and that Rac2 was key for NADPH oxidase assembly in CD8+ DCs (53). The small GTPase Rab27a was also necessary for NAPDH oxidase assembly (54). However another group, using slightly different conditions, found that while NOX2 did indeed reduce phagosomal proteolysis, this was not associated with significant changes in phagosomal pH. Rather, this group proposed that in DCs and macrophages, NOX2 affects proteolysis through reversible inhibition of the action of cysteine cathepsins *via* H_2_O_2_-driven oxidation of cysteine residues. Aspartic cathepsins are unaffected by the presence of NOX2 and thus the phagocyte NADPH oxidase was proposed to alter the activity of only a subset of proteases, skewing the peptide repertoire generated (55, 56).

A recent publication by Reis e Sousa and colleagues provided an intriguing new insight into the role of NOX2-derived ROS in antigen presentation (57). The DNGR1 receptor, expressed on the conventional DC1 (cDC1) subset of DCs, is essential for effective cross-presentation. DNGR1 binds F-actin on dead cell corpses, and has a short hemi-ITAM motif that can recruit and activate Syk. Mice that are deficient in DNGR1 or Syk expression in DCs have impaired cross presentation (58).

This group demonstrated that DNGR1 ligation facilitates Syk kinase activation and this, in turn, leads to NOX2 activation within phagosomes containing internalised antigen. The oxidative stress caused by the resulting free radicals damages the phagosome, causing membrane rupture, thus allowing leak of antigen into the cytosol and its translocation into the MHC class I presentation pathway. Cross presentation is markedly impaired in gp91phox-deficient DCs. How exactly Syk drives NOX2 activation in this context is unknown but the NOX2 activators Vav1 and Rac have previously been shown to be necessary for efficient cross-presentation and they are likely to be involved to some extent.

### 2.2 MHC Class II Processing and Presentation

There is also evidence that NOX2 can influence MHC class II peptide processing and presentation. Extending their previous work on cysteine cathepsins Yates and colleagues demonstrated that NOX2 not only affects the amount of proteolytic processing but affects it qualitatively too (59). They used the myelin oligodendrocyte glycoprotein (MOG) -induced model of experimental autoimmune encephalomyelitis (EAE) and showed that NOX2-derived macrophages (though interestingly not DCs) were defective in their ability to process and present the I-A(b)-immunodominant peptide of MOG. As such, p47*phox* or gp91*phox* deficient mice were partially protected from the central nervous system injury and inflammation that characterises EAE. MHC class II presentation is also impaired in human B cells deficient in p40*phox* (60). This was most marked for cytoplasmic and endogenous antigen but processing of membrane antigen was normal (see *Altered Humoral Immune Response in NOX2 Deficiency* below).

## 3 NOX2 in Regulating Adaptive Immunity

Although most commonly associated with innate immunity, NOX2 also has a variety of signalling roles in T and B cell responses. Some of these include; modulating T helper differentiation, proliferation of B cells and inducing apoptosis.

### 3.1 NOX2 in CD4+ T Cells

#### 3.1.1 NOX2 Signalling Influences CD4+T Helper Differentiation

There are many conflicting studies regarding the influence of NOX2 on the differentiation of T helper subsets, summarised in **Table 1**. Briefly, the first published study on T helper differentiation in NOX2 deficiency describes a preferential Th1 response in NOX2^-/-^ CD4+ T cells (61). Secretion of IFNγ is typical of a Th1 response, IL-4 and IL-5 of a Th2 response, and IL-17 and TGFβ of a Th17 response. Jackson et al. (61) found an increase in IFNγ secretion and decrease in IL-4 and IL-5 secretion following stimulation with anti-CD3. However, Kwon et al. found an increase in IL-4 secretion following anti-CD3 and anti-CD28 stimulation, indicative of a Th2 response in NOX2 deficiency (62). In opposition to both, Tse et al. (63) describe that a Th17 response develops in NOX2^-/-^ CD4+ T cells following anti-CD3 and anti-CD28 stimulation. They found decreased IFNγ and IL-4 secretion, but increased IL-17 and TGFβ secretion (63). Although, this group used the NOD strain of mice that are renowned for their autoimmune phenotype, whereas the studies mentioned previously used the C57BL/6 strain, which may account for the difference in findings. Most studies find a combined Th1/Th17 response in NOX2 deficiency, with increased levels of IFNγ, IL-17 and their associated transcription factors T-bet and RORγt (64–66). Interestingly, Lee et al. (60) found that under their specific polarising conditions, the differentiation of all T helper subsets was elevated when NOX2 was absent, demonstrating that ROS are required to balance the development of T cell responses. The importance of NOX2 in CD4+ T helper differentiation remains to be clarified, but it appears that proinflammatory Th1/Th17 skewing is favoured. There are currently no published studies on the effect of NOX2 deficiency on the differentiation of Th9 or T follicular helper cells (Tfh). It would be both interesting and important to understand the entire T cell phenotype in the context of NOX2 deficiency.

**Table 1:** Summary of studies describing T helper differentiation in NOX2 deficiency.

Skew	Cytokine / transcription factor altered	Antigen	Strain	Gene deleted	Ref
Th1	↑ IFNγ ↓ IL-4 ↓ IL-5	Anti-CD3	C57BL/6	p47*phox* gp91*phox*	(56)
Th2	↑ IL-4	Anti-CD3 + anti-CD28	C57BL/6	gp91*phox*	(57)
Th17	↓ IFNγ ↓ IL-4 ↑ IL-17 ↑ TGFβ	Anti-CD3 + anti-CD28	NOD	p47*phox*	(58)
Th1/Th17	↑ IFNγ ↑ IL-17 ↓ IL-4 ↓ IL-5 ↑ T-bet ↓ GATA-3	Anti-CD3 + anti-CD28	C57BL/6	gp91*phox*	(59)
Th1/Th17	↑ IFNγ ↑ IL-17 ↑ RORγt	PMA + ionomycin	C57BL/6	gp91*phox*	(60)
Th1/Th17	↑ IFNγ ↑ IL-17	*In vivo* OVA challenge *In vitro* anti-CD3 + anti-CD28	C57BL/6	gp91*phox*	(61)

#### 3.1.2 NOX2 Signalling Affects Treg Differentiation and Activity

NOX2 is involved in the differentiation of other T cell types, including T regulatory cells (Tregs). One study found fewer peripheral CD4+CD25+ Tregs and decreased FOXP3 expression in NOX2^-/-^ mice, indicating that NOX2 derived ROS also play a role in controlling the development of Tregs (65). However, a recent study found no decrease in Treg number or function in CGD patients, except in those with X-linked gp91*phox* deficiency (67). The authors suggest this may coincide with the fact that gp91*phox*^-/-^ CGD patients have more inflammatory symptoms than those with mutations in other NADPH oxidase subunits (49, 67).

NOX2 is required for restraining the expression of the immune suppressive molecules on Tregs. The expression of CTLA-4, GITR, CD39 and CD73 is significantly greater on gp91*phox*^-/-^ Tregs. Additionally, gp91*phox*^-/-^ Tregs have increased NF-kB activation and greater FOXP3 expression. Subsequently, Tregs deficient in gp91*phox* have greater suppressive activity than wildtype control Tregs (68). Interestingly, p47*phox*^-/-^ Tregs have poorer suppressive capabilities compared to their wildtype counterparts (69). This may relate to the functions of p47*phox* independent of phagocyte NADPH oxidase (70, 71).

#### 3.1.3 NOX2 Is Required for T Cell Apoptosis

NOX2 is required for inducing cell-intrinsic apoptosis in activated T cells during the resolution of an immune response (72). Apoptosis of excess T cells after antigen clearance is essential to prevent an over exuberant immune response when responding to new and repeated antigenic challenges. gp91*phox*^-/-^ T cells display significantly improved survival *in vivo* following cytokine deprivation. Greater antigen-specific proliferative responses are also observed when compared to wildtype controls, due to the larger pool of T cells that remain after the initial antigen challenge (72). This increased T cell survival may account for the differences in cytokine secretion discussed in section *NOX2 Signalling Influences CD4+T Helper Differentiation*.

### 3.2 NOX2 in CD8+ T Cells

#### 3.2.1 NOX2 Signalling Can Affect CD8+ T Cell Responses

CD8+ T cell responses are critical to eliminate intracellular pathogen infections. In the absence of NOX2, mice are highly susceptible to *Trypanosoma cruzi* infection. There are fewer CD8+ T cells present at baseline in p47*phox*^-/-^ mice, and these fail to proliferate in response to *T. cruzi* infection (73). Conversely, p47*phox*^-/-^ CD8+ T cells have improved survival and mice experience reduced viral titres in response to lymphocytic choriomenigitis virus (LCMV) infection. The authors state this improved CD8+ T cell viral response may be due to less immunopathology that occurs in the absence of p47*phox* (74). Similarly, gp91*phox*^-/-^ mice have reduced inflammation and viral titres in response to influenza infection, however there was no difference in CD8+ T cell populations *in vivo* or influenza-specific CD8+ T cell responses *in vitro* (75). Therefore, the influence of NOX2 on CD8+ T cell responses may be dependent upon pathogen type.

#### 3.2.2 NOX2 Is Critical for CD8+ Treg Driven Immunosupression

NOX2 is utilised by CD8+ Tregs to enable a novel Treg mediated suppression of CD4+ T cells (76). CD8+ Tregs are thought to release exosomes containing NOX2, which is taken up by CD4+ T cells located in nearby T cell zones of secondary lymphoid organs. NOX2 derived ROS inhibits the phosphorylation of the T cell receptor (TCR) signalling molecules ZAP70 and LAT, inhibiting TCR signal transduction. CD8+ Tregs treated with the flavoenzyme inhibitor diphenyleneiodonium (DPI), gp91ds-tat or short hairpin RNAs targeting NOX2 are unable to upregulate NOX2 and subsequently are unable to supress CD4+ T cell activation (76).

### 3.3 NOX2 in B Cells

#### 3.3.1 NOX2 Elicits Bacterial Killing in B Cells

Similar to innate immune cells but unlike T cells, peritoneal B cells can utilise NOX2 derived ROS to kill intracellular bacteria. NOX2^-/-^ B cells from NOX2 deficient mice have a reduced ability to produce the ROS required to kill engulfed pathogens, and therefore have greater survival of bacteria within phagosomes (77).

#### 3.3.2 NOX2 Signalling Restrains Proliferation of B Cells

Following B cell receptor (BCR) stimulation, NOX2 is responsible for generating the rapid initial production of ROS, whereas the later stages of ROS production are not NOX2 dependant. NOX2^-/-^ mice fail to produce ROS immediately after BCR stimulation, but BCR proximal signalling and subsequent downstream signalling pathways are normal (78, 79). However, NOX2^-/-^ B cells have been found to undergo enhanced cell cycle entry following BCR stimulation (79, 80). This suggests NOX2 has a role in negatively modulating ROS-driven BCR induced proliferation in B cells.

#### 3.3.3 NOX2 Is Involved in B Cell Signalling

NOX2 derived ROS often acts as a second messenger during various signalling pathways. Tyrosine phosphorylation and IgM secretion is impaired following BCR or TLR4 stimulation in NOX2 deficient B cells. Accordingly, lentiviral induced expression of NOX2 components can restore signalling capabilities in NOX2 deficient cells following BCR stimulation (81). NOX2^-/-^ B cells have increased expression of the Toll-like receptors (TLR) TLR7 and TLR9, and subsequently have greater responsiveness to TLR7/9 stimulation (82). These studies demonstrate that NOX2 can modulate BCR signalling in a number of ways.

#### 3.3.4 NOX2 Regulates MHC Class II Antigen Presentation on B Cells

Presentation of exogenous antigens requires antigen uptake and processing in endosomal or lysosomal compartments to generate the peptides to be presented on MHC class II molecules [reviewed in (83)]. p40*phox*^-/-^ B cells are less able to present exogenous antigen on their MHC class II. However, p40*phox*^-/-^ B cells preferentially present self-membrane resident antigens, suggesting p40*phox* may skew epitope selection and have implications for CD4+ T cell activation (60).

#### 3.3.5 Altered Humoral Immune Response in NOX2 Deficiency

NOX2 may have a role in the production of antibodies. NOX2^-/-^ mice have greater antibody production following injection of collagen (84, 85) and challenge with UV-irradiated bacteria (86). Cachat et al. (88) found an increase in IgG1 and IgG2c production in NOX2^-/-^ mice following ovalbumin injection. A later paper found NOX2^-/-^ mice have increased production of IgA, IgG, IgG1, IgG2b and IgG3 levels following influenza A infection (88). The authors suggest that functional NOX2 activation during influenza A infection results in the suppression of antiviral cytokines, preventing the development of humoral immunity (88). Interestingly, there may be some differences between human and mouse. IgG1 levels are decreased whereas IgG2 levels are increased in CGD patient serum (87). CGD patients also have significantly increased levels of B cell activating factor (BAFF), a B cell survival factor, and subsequently have greater IgM levels compared to healthy controls (89). CGD patients have decreased numbers of influenza-specific peripheral memory B cells but increased numbers of nonconventional CD27- memory B cells compared to healthy controls (90, 91). Although, despite abnormal numbers of B memory cells, influenza specific memory B cell responses remain comparable to healthy controls (90). Therefore, NOX2 is involved in inducing and maintaining the humoral immune response, however the specific role of NOX2 in human B cell responses needs to be investigated further.

## 4 The Deleterious Role of ROS in Systemic Inflammation

In the sections above, we have seen that ROS are pivotal for both normal innate and adaptive immunity. We have also described how a lack of ROS in CGD can lead to autoinflammation and autoimmunity.

However, we have also seen how the generation of ROS must be tightly controlled and its generation can outstrip the capability of those systems that regulate it. Excess ROS can cause tissue damage in a variety of ways, causing protein and DNA damage and lipid peroxidation.

### 4.1 Oxidative Stress in Systemic Disease

Oxidative stress is well known to be a contributing factor in the development of neurodegenerative diseases such as Alzheimer’s disease, Parkinson’s disease and Multiple Sclerosis. Dysregulation or overproduction of ROS leads to oxidative stress which is thought to disrupt immune homeostasis in the central nervous system (CNS) and promote prolonged neuroinflammation (92). NOX proteins are important generators of ROS in the CNS and NOX2 expression has been documented in the CNS in microglia, neurons and endothelial cells (93).

## 5 Reactive Oxygen Species in the CNS

### 5.1 Amyotrophic Lateral Sclerosis

Amyotrophic lateral sclerosis (ALS) is a fatal neurodegenerative disease characterised by the progressive loss of motor neurons in the brain, brain stem and spinal cord. Disease progression is rapid, with a prognosis of only 2-5 years after diagnosis for most individuals (94).

Several studies have demonstrated that NOX2 contributes to disease progression in the SOD1^G93A^ transgenic mouse, a common mouse model of ALS. NOX2 expression and activation was shown to be significantly upregulated in microglia in the spinal cord of SOD1^G93A^ mice compared to controls (95). NOX2 expression was also found to be increased in the spinal cord of sporadic ALS patients. The authors demonstrated that NOX2 deletion in SOD1^G93A^ transgenic mice prolonged survival and slowed disease progression, suggesting that NOX2 activity contributes to the degeneration of motor neurons and disease progression in ALS. Another study demonstrated that treatment of SOD1^G93A^ mice with apocynin, a NOX inhibitor, also increased survival and slowed disease progression (96). The authors also demonstrated that SOD1 regulated RAC1/NOX2 dependent ROS generation in a redox-dependent manner. ALS-associated SOD1 mutants resulted in enhanced activation of RAC1/NOX2 and increased ROS production in both cell lines and the spinal cord of SOD1^G93A^ mice.

However, in contrast to this, a recent study found that NOX2 inhibition did not extend survival in SOD1^G93A^ mice (97). Deletion of NOX1 or NOX2 in SOD1^G93A^ mice did not increase survival or influence microglia activation in this study. Treatment of SOD1^G93A^ mice with the NOX inhibitors thioridazine and perphenazine did not significantly increase survival or prevent motor neuron degeneration. A study of the oxidative burst in granulocytes in the peripheral blood of ALS patients did not identify any significant difference in NOX2 activity between ALS patients and matched controls. However, patients with lower NOX2 activity were found to have a significant increase in survival (98). Therefore, whilst NOX2 has been shown to be upregulated in both mouse models and patients with ALS, the exact role of NOX2 in ALS pathogenesis remains unclear.

### 5.2 Multiple Sclerosis

Multiple sclerosis (MS) is a common neurodegenerative disorder characterised by inflammation and demyelination in the CNS. Microglial activation is hypothesised to play an important role in the pathogenesis of MS. NOX2 has also been shown to be upregulated in microglia in active lesions in MS patients (99).

The mouse experimental autoimmune encephalomyelitis (EAE) model is commonly used as a model to study MS. Recent evidence has demonstrated that disease severity is reduced in NOX2^-/-^ mice after EAE induction. Lymphocyte and microglial infiltration in the CNS was also significantly decreased compared to heterozygous and wild-type controls. Interestingly, the authors showed that only immune infiltration in the CNS is reduced, as immune cell populations in peripheral tissues such as the spleen and cervical lymph node are similar in NOX2^-/-^ and wild-type mice post EAE induction (100). Microglial activation was also decreased in NOX2^-/-^ mice, with inflammatory cytokine and chemokine secretion levels in the CNS also decreased.

Another recent paper demonstrated that deletion of NOX2 in conventional DCs (cDCs) reduced disease severity and demyelination in an adoptive transfer model of EAE (101). Interestingly, the authors demonstrated that deletion of NOX2 in cDCs reduced accumulation and activation of autoimmune CD4+ T cells in the CNS in EAE mice, suggesting that NOX2 regulates CD4 infiltration. Deletion of NOX2 also abrogated LC3-associated phagocytosis and CD4+ T cell activation through reduced myelin antigen presentation. This study highlights an important role for NOX2 in promoting inflammation and demyelination in EAE mice. Therefore targeting NOX2-dependent ROS production may slow disease progression and provide therapeutic benefit for patients with MS.

### 5.3 Alzheimer’s Disease

Oxidative stress and damage have also been hypothesised to play a role in Alzheimer’s disease, although the role of NADPH oxidases remains unclear. As mentioned previously, microglia play a vital immune role in CNS homeostasis through clearance of dead cells and debris. However, dysregulation of microglia can lead to prolonged neuroinflammation and the development of neurodegenerative disorders. Microglia have been shown to associate with and promote clearance of amyloid-β (Aβ) deposits in the early stages of Alzheimer’s. However, in aging mice microglia appear to have a reduced ability to clear Aβ deposits and drive inflammation within the CNS (102).

NOX2 expression in microglia has also been hypothesised to play an important role in the pathogenesis of Alzheimer’s disease. A recent study demonstrated high NOX2 expression in microglia and increased microglial infiltration in aged wild-type brains, compared to young mice (103). Interestingly, NOX2*^-/-^* aged mice had significantly less Aβ deposition and plaque formation compared to aged wild-type controls. ROS production was also much lower in NOX2*^-/-^* mice than in wild-type mice, indicating that ROS production in the aged mice was NOX2-dependent. The authors also investigated ROS production in human brain tissue, and found older individuals had higher levels of ROS production when compared to young controls. Stimulation of the BV2 microglial cell line with Aβ_42_ peptide also resulted in significantly increased NOX2-depdendent ROS production, which could be inhibited using NOX2 inhibitors such as apocynin or Nox2tat. These results indicate that NOX2 may play an important role in the regulation of microglia and the microglial response to Aβ plaques and therefore it may be an important driver of the pathogenesis of Alzheimer’s disease.

Whilst it is clear that oxidative stress is involved in aging and the development of neurodegenerative diseases, the precise mechanisms defining how aberrant NOX2-depdendent ROS production drives neuroinflammation require further investigation. In addition, it remains to be investigated whether targeting of NOX2 through the use of inhibitors would provide therapeutic benefit in neurodegenerative disorders.

## 6 The Role of Reactive Oxygen Species in the Lung

ROS production by phagocytes plays a vital role in the innate immune response and the clearance of pathogens during infection. However, it is essential that the mechanisms which regulate ROS generation are tightly controlled. Failure to regulate the innate immune response results in excessive ROS production, or oxidative stress, which promotes inflammation. Oxidative stress and the resultant sustained inflammation can result in tissue damage, particularly in barrier sites such as the lung (104). Recent evidence has implicated excessive NOX2-derived ROS production in acute lung injury, particularly during influenza infection.

In 2006, Snelgrove et al. demonstrated that deletion of NOX2 in mice resulted in reduced viral load after infection with Influenza virus (105). NOX2^-/-^ mice exhibited enhanced viral clearance, increased lung function and reduced lung damage when compared to wild-type mice. Increased macrophage and neutrophil infiltration into the airway epithelia was also observed. Another study by Vahlos et al. in 2011 also demonstrated that deletion of NOX2 resulted in reduced viral titres in mice infected with Influenza A virus (75). Apoptosis of lung alveolar epithelial cells was greatly reduced in lung tissue sections of NOX2*^-/-^* mice compared to wild-type mice. Interestingly, in contrast to the earlier findings by Snelgrove et al., the authors demonstrated that immune cell infiltration in the bronchoalveolar lavage fluid (BALF) was significantly decreased in NOX2*^-/-^* mice. However, the authors hypothesise that this could be due to sex differences between the mouse models used in the studies. Treatment of wild-type mice with the ROS inhibitor apocynin after Influenza A infection also significantly reduced macrophage and neutrophil infiltration and viral titres were reduced by 50%. These results indicate that NOX2 is driving inflammation and acute lung injury in response to Influenza A infection. Therefore, modulation of NOX2-dependent ROS production may provide therapeutic benefit and reduce lung damage in patients suffering from acute lung injury during infection.

A recent study identified that Influenza A infection drives the production of endosomal NOX2-dervied ROS in response to TLR7 stimulation by viral RNA (88). Endosomal ROS was also found to suppress cytokine secretion in a TLR7-dependent manner. Treatment with apocynin significantly increased IL-1β, TNF-α, IFN-β and IL-6 secretion in wild-type macrophages in response to imiquimod. Interestingly, the authors identified a single cysteine residue, Cys98, which is highly conserved and unique to TLR7, as a novel redox sensor. TLR7^-/-^ macrophages transfected with a TLR7C98A mutant could not restore TLR7-dependent cytokine secretion. The authors hypothesise that ROS production by NOX2 may modify the Cys98 residue, resulting in reduced cytokine secretion and a dampened antiviral response (88). In Nox2 deficient mice infected with Influenza A virus, IL-1β and IFN-β secretion was significantly increased. Serum and BALF levels of IgG and IgA were also significantly increased compared to wild-type mice, indicating that NOX2-derived ROS can also suppress antibody production. These results indicate that ROS production can inhibit important antiviral responses, thereby reducing the host’s ability to efficiently clear viral pathogens.

Recent evidence has also demonstrated that NOX2 can modulate Type I Interferon (IFN) production in response to bacterial infections. NOX2^-/-^ mice infected with *Listeria monocytogenes* exhibited an increased bacterial load, whereas *Ifnar1^-/-^* mice infected with *L. monocytogenes*. had a reduced bacterial load (106). Deletion of NOX2 in *Ifnar1^-/-^* mice also resulted in a reduced bacterial load, indicating that NOX2 regulation of Type I IFN controls *L. monocytogenes* infection. The number of infection foci was increased in NOX2^-/-^ mice, however lymphocyte migration to infection foci was decreased in a Type I IFN-dependent manner. Interestingly, the authors also demonstrated that NOX2 deficiency upregulates IL-10 expression, which is known to play an anti-inflammatory role during infection. These results suggest a novel antimicrobial role for NOX2 in controlling *L. monocytogenes* infection through modulation of the Type I IFN response.

## 7 Conclusion

**Figure 2:**
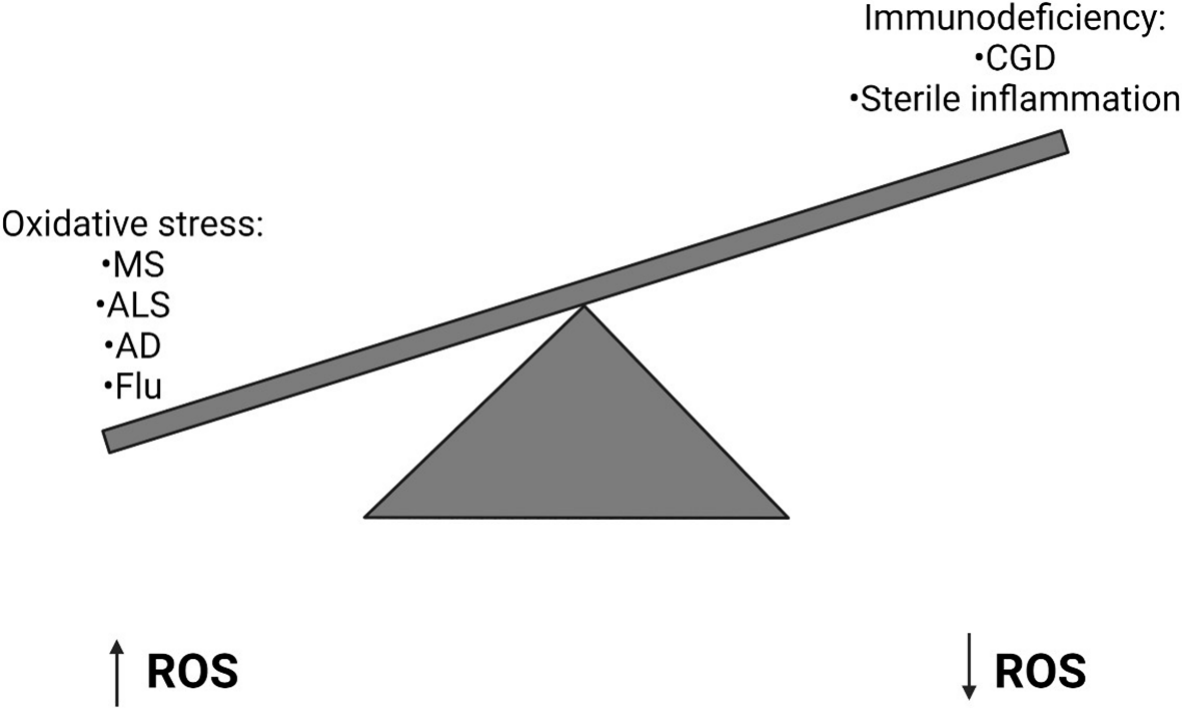
Imbalanced Reactive Oxygen Species generation can have implications for human health. High levels of Reactive Oxygen Species (ROS) can result in oxidative stress, which can lead to a number of diseases including Multiple Sclerosis (MS), Amyotrophic Lateral Sclerosis (ALS), Alzheimer’s Disease (AD) and Influenza. Alternatively in primary immunodeficiencies, such as Chronic Granulomatous Disease (CGD), where a genetic defect means ROS are not generated, sterile inflammation often develops.

The balanced generation of ROS is essential to induce appropriate immune responses and avoid tissue damage by oxidative stress (**Figure 2**). ROS is required during the innate immune response to control invading pathogens and prevent infection. Too little ROS results in susceptibility to opportunistic pathogens, such as in CGD. However, increasing evidence has demonstrated that dysregulation of ROS production can result in sustained inflammation and tissue damage which can be fatal during severe infection. Recent evidence has also demonstrated that ROS can prevent antiviral immunity and reduce the body’s ability to clear viral infections. Therefore, inhibition of NOX2 during infection may help to promote antiviral responses and prevent excessive ROS generation, thereby reducing the occurrence of acute lung injury, for example in Influenza infection. It is important to further investigate the mechanisms underlying the regulation of ROS generation and how they become dysregulated during infection in order to understand how they may be targeted for clinical benefit in the future. Beyond influenza A, the utility of blocking NOX2 action should be examined in other settings such as acute lung injury and Acute Respiratory Distress Syndrome (ARDS). The question of blocking host inflammatory pathways in infection related lung pathology has been shown into sharp focus by the COVID-19 pandemic where treatment with dexamethasone (107) or IL-6 inhibition (108) can have a beneficial effect when used at the correct stage of the disease. It will be interesting to see if NOX2 inhibition has any beneficial effect in either *in vitro* or animal models of COVID-19 as it is likely that the virus will continue to mutate and become endemic, possibly escaping vaccine-mediated control in some instances.

Numerous NOX2 inhibitors have been developed (109) and it is likely that advances in *in silico* technology such as alpha-fold will also better inform our efforts to inhibit the action of this and other NADPH oxidases (110, 111).

## Author Contributions

PM, SM, and DT: conceptualisation and writing. DT: funding acquisition and final revision. All authors contributed to the article and approved the submitted version.

## Funding

DT is funded by a Wellcome-Beit Prize Trust Clinical Research Career Development Fellowship (grant code 098051) and Imperial College London. Research in DT lab is supported by a generous donation from the family of Sidharth Burman.

## Conflict of Interest

The authors declare that the research was conducted in the absence of any commercial or financial relationships that could be construed as a potential conflict of interest.

## Publisher’s Note

All claims expressed in this article are solely those of the authors and do not necessarily represent those of their affiliated organizations, or those of the publisher, the editors and the reviewers. Any product that may be evaluated in this article, or claim that may be made by its manufacturer, is not guaranteed or endorsed by the publisher.
